# Advancing Remote Life Sensing for Search and Rescue: A Novel Framework for Precise Vital Signs Detection via Airborne UWB Radar

**DOI:** 10.3390/s25175232

**Published:** 2025-08-22

**Authors:** Yu Jing, Yili Yan, Zhao Li, Fugui Qi, Tao Lei, Jianqi Wang, Guohua Lu

**Affiliations:** Department of Military Biomedical Engineering, Air Force Medical University, Xi’an 710032, China; jingyu1998@fmmu.edu.cn (Y.J.); richard1207@163.com (Y.Y.); lizhaofmmu@fmmu.edu.cn (Z.L.); qifg1992@fmmu.edu.cn (F.Q.); leitaoxman@fmmu.edu.cn (T.L.); wangjq@fmmu.edu.cn (J.W.)

**Keywords:** airborne radar, vital signs detection, remote sensing, search and rescue

## Abstract

**Highlights:**

**What are the main findings?**

- An airborne bio-radar system to remotely sense vital signs of survivors for post-disaster search and rescue was developed.
- Theoretical analysis of the impact of interference coming from the motion of the UAV platform and echoes from the background environment on radar detection performance.

**What is the implication of the main finding?**

- A signal processing framework based on blind source separation was proposed to precisely extract the respiration and heartbeat, which combines the high-order analytical tool and the feedback notch filter.
- The remote high-resolution vital signs detection approach is suitable for real-world applications such as search and rescue.

**Abstract:**

Non-contact vital signs detection of the survivors based on bio-radar to identify their life states is significant for field search and rescue. However, when transportation is interrupted, rescue workers and equipment are unable to arrive at the disaster area promptly. In this paper, we report a hovering airborne radar for non-contact vital signs detection to overcome this challenge. The airborne radar system supports a wireless data link, enabling remote control and communication over distances of up to 3 km. In addition, a novel framework based on blind source separation is proposed for vital signals extraction. First, range migration caused by the platform motion is compensated for by the envelope alignment. Then, the respiratory waveform of the human target is extracted by the joint approximative diagonalization of eigenmatrices algorithm. Finally, the heartbeat signal is recovered by respiratory harmonic suppression through a feedback notch filter. The field experiment results demonstrate that the proposed method is capable of precisely extracting vital signals with outstanding robustness and adaptation in more cluttered environments. The work provides a technical basis for remote high-resolution vital signs detection to meet the increasing demands of actual rescue applications.

## 1. Introduction

Wild search and rescue (SAR) is one of the emphases and difficulties of current emergency rescue medicine [1]. In harsh post-disaster environments, the road is damaged, the traffic is interrupted, the disaster area is vast, and the distribution of the survivors is unknown, which will pose challenges to the deployment of SAR work [2]. In recent years, unmanned aerial vehicles (UAV) have been gradually used to assist rescue of the trapped hikers and missing persons, and some incidents have shown the specific capability and superiority of the UAVs in large-scale disaster risk reduction due to its flexibility, mobility, and no casualties [3,4,5].

At present, sensors equipped by a UAV-based SAR system mainly include optical sensors (e.g., high-resolution camera [6,7,8], thermal imaging camera [9,10,11], and multi-spectral camera [12,13,14]) and radio frequency sensors (e.g., bio-radar [15,16]). The principles, advantages, and applicable scenarios of these sensors are different. The optical sensors identify targets based on the morphological features, the optical features, and the infrared radiation energy of the human body. However, optical sensors are susceptible to interference from environmental conditions, such as temperature, smoke, fog, and obstruction. Bio-radar senses the presence and physiological activities of humans by transmitting electromagnetic waves and analyzing the received echoes, which is the most effective way to detect vital signs under the above conditions.

**Figure 1:**
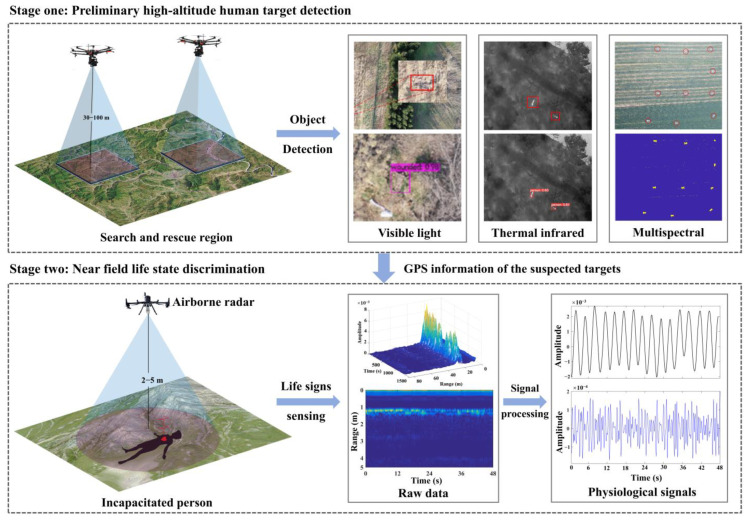
Two-stage unmanned search and rescue scheme.

Based on this, our previous study proposed an unmanned SAR scheme based on multi-source sensors [17], which involves two stages. First, the three optical camera-based UAV systems were released to detect human targets and acquire their position information through global positioning system (GPS) by scanning the disaster-affected area. Second, the radar-based UAV system proceeds to the corresponding positions and hovers to sense physiological information of human targets. In addition, the radar-based UAV system could also find the unconscious victim under dense smoke or under the ruins as shown in Figure 1, which would provide the scientific basis for the formulation of SAR strategies and the allocation of rescue resources.

However, two challenges make the vital signs detection problem of the airborne radar system difficult. First, the UAV platform will inevitably generate relative movement, and human physiological signals are comparatively weak, which will be overwhelmed by the platform motion [18]. Second, the background clutter from the environment where the human target is located will further interfere with the radar echoes. It means that in order to detect vital signs with this technology, we need to precisely separate the wanted signals from the aforementioned multiple interferences.

In recent years, several methods have been proposed to address the platform motion compensation problem. In [18], Cardillo et al. first extracted the radar self-motion from the signals reflected by stationary objects through identifying the clutter range without any additional sensor. In [19], Rong et al. proposed a method based on phase residual, which compensates the platform motion by calculating the residual phase between the human subject and the static background, and then decomposing the residual for vital signals extraction. In [20], Zhang et al. proposed an algorithm to extract respiratory information of the victims by segmenting the UAV motion into multiple time intervals and computing the average energy ratio of the UAV motion signal and human echo signal in the selected frequency band to estimate the compensation coefficient. In [21], a rotating radar UAV system was designed to locate the trapped individuals in collapsed buildings, and a new unwrapping algorithm was proposed to estimate and compensate for the UAV motion based on the reflected echoes. However, the methods mentioned above are mostly for indoor location and based on signals reflected by stationary objects, and the performance may degrade when there is no stationary object. Furthermore, the impact of the background clutter where the human target is located on the detection performance of the airborne radar system has not been thoroughly investigated.

In this paper, a portable airborne radar vital signs sensing system is developed for remote data collection, which adopts impulse–radio ultra-wide band (IR-UWB) radar and Mesh network technology. To address the aforementioned problems, the effect of the platform motion and the background noise on radar detection capacity are theoretically analyzed. Then, a signal processing method based on blind source separation is proposed to separate out vital signals from multiple interferences. First, range migration caused by the platform motion is coarsely compensated by the adjacent envelope alignment method based on cross correlation. Second, we analyze the statistical characteristics of the measured dynamic background clutter from the grassland scenario and find that it may obey the Gaussian distribution, or the mixed Gaussian distribution. In other words, it can be regarded as a Gaussian noise. Then, the respiratory waveform of the human target is extracted by the joint approximative diagonalization of eigenmatrices (JADE) algorithm, which is based on higher-order statistical analysis to remove Gaussian components. Finally, the heartbeat signal is recovered by respiratory harmonic suppression through a feedback notch filter. The performance of this method has been evaluated and tested by field experiments in different scenarios, and these results confirm that the proposed method improves the performance of airborne radar system without additional sensors.

The remainder of this paper is organized as follows. The airborne radar system composition is introduced in Section 2. The signal model is described in Section 3. The proposed method is deliberated in Section 4. The experimental setup and results are presented in Section 5. Finally, the conclusions and future works are given in Section 6.

## 2. Airborne Bio-Radar System Design

**Figure 2:**
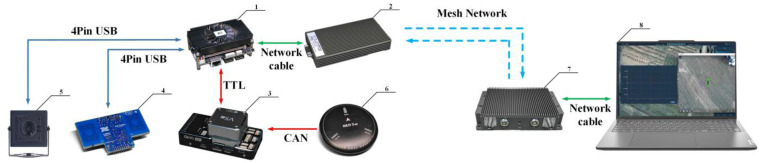
Hardware structure of airborne radar system. (1). Onboard computer. (2). Transmission radio station of data collection end. (3). Flight controller. (4). X4M200 IR-UWB radar. (5). High-definition camera. (6) GPS module. (7) Transmission radio station of data reception end. (8) Ground station for visualizing.

This section generally introduces the hardware and software design of the portable airborne radar system. Figure 2 presents the hardware structure block diagram of the system, which consists of onboard end and ground receiving end. The hardware components of the onboard end include the sensor module, data processing center, and data transmission module. The ground receiving end is utilized to visualize sensor data and information of the casualty on the map.

The quadcopter UAV is equipped with three types of sensors: X4M200 IR-UWB radar, high-definition camera, and GPS. The HZHY-AI313 UAV onboard computer drives the three sensors for data acquisition, and it can process data simultaneously. Then, the collected data are transmitted to the ground receiving end through a Mesh network constructed by image transmission radio stations [22].

The software control program of the system is developed based on the topic and service architecture of ROS2 (Robot Operating System). First, the ground control station operates the UAV to take off and runs the MAVROS program to obtain real-time position and attitude information of the UAV. After the UAV arrives at the search area, it hovers above the designated location, and then the sensor-driven node is operated to activate the UWB radar and visible light camera to recognize and perceive the injured target. Finally, the data transmission node transmits the position and attitude information of the UAV, the physiological information of the target, and the image information to the ground control station for further processing.

**Figure 3:**
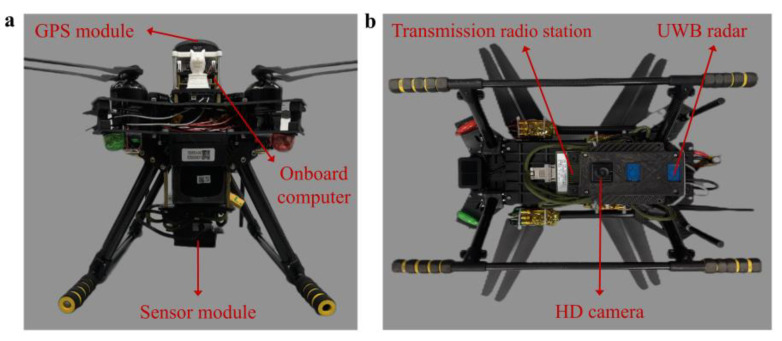
Pictures of airborne radar system. (**a**) Front view of the system. (**b**) Bottom view of the system.

The pictures of the airborne radar system are shown in Figure 3. Its weight is about 3 kg, and its endurance of flight is about 25 min. Its communication distance is up to 3 km in an unobstructed environment without any data package loss.

## 3. Sensing Model

### 3.1. Principle of UWB Radar for Vital Signs Detection

**Figure 4:**
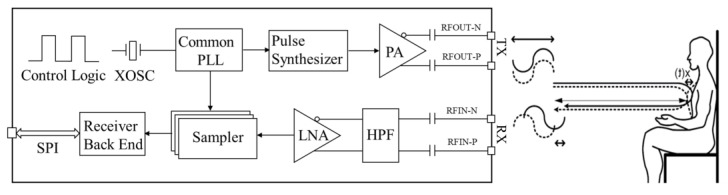
Block diagram and detection principle of X4M200 IR-UWB radar.

Figure 4 shows the block diagram and detection principle of a typical UWB radar, which measures the physiological activities of a human target by demodulating the received signal phase [23]. The response of radar can be expressed as:

(1) $$h\left(t,\tau \right)=a_{v}\delta \left(\tau -\tau_{v}\left(t\right)\right)+\sum_{i}a_{i}\delta \left(\tau -\tau_{i}\right)$$

where $a_{v}\delta \left(\tau -\tau \left(t\right)\right)$ is the response of the human target, $\sum_{i}a_{i}\delta \left(\tau -\tau_{i}\right)$ is the response of other static clutter.

Assuming that there is a stationary human target at a distance $d_{0}$, when radar is mounted on a mobile platform to detect the human target, the instantaneous distance from the antenna to the surface of the human chest cavity can be expressed as [16]:

(2) $$d\left(t\right)=d_{0}+A_{r}\sin\left(2\pi f_{r}t\right)+A_{h}\sin\left(2\pi f_{h}t\right)+d_{UAV}\left(t\right)$$

where $A_{r}$ and $f_{r}$ are amplitude and frequency of respiratory signal, $A_{h}$ and $f_{h}$ are amplitude and frequency of heartbeat signal. $d_{UAV}$ is the platform motion. In this scenario, the delay of a static object will be dynamic; the response of radar can be further expressed as [24]:

(3) $$h\left(t,\tau \right)=a_{v}\delta \left(\tau -\tau_{v}\left(t\right)\right)+a_{s}\delta \left(\tau -\tau_{s}(t)\right)$$

Time delay $\tau_{v}\left(t\right)$ of human target is equal to:

(4) $$\tau_{v}\left(t\right)=\frac{2d\left(t\right)}{c}$$

Time delay $\tau_{s}(t)$ of a static object is equal to:

(5) $$\tau_{s}\left(t\right)=\frac{2{[d}_{0}\left(t\right)+d_{UAV}\left(t\right)]}{c}$$

where c is the speed of transmitted signal. The received signal can be expressed as:

(6) $$R\left(t,\tau \right)=s\left(\tau \right)*h\left(t,\tau \right)=a_{v}s\left(\tau -\tau_{v}\left(t\right)\right)+a_{s}s\left(\tau -\tau_{s}\left(t\right)\right)$$

where $s\left(\tau \right)$ is the radar transmitted pulse.

The expression function of radar echo signal of human target is:

(7) $$S\left(t\right)=Ae^{j\left(2\pi f_{c}t+\theta \left(t\right)+\varphi_{0}\right)}$$

where $f_{c}$ equals to 7.29 GHz and $\varphi_{0}$ is the initial phase, the phase signal can be calculated as [25]:

(8) $$\theta \left(t\right)=\frac{4\pi }{\lambda }(X\left(t\right)+d_{UAV}(t))=\frac{4\pi }{\lambda }X\left(t\right)+\frac{4\pi }{\lambda }d_{UAV}\left(t\right)$$

where λ is wavelength of transmitted signal, $X\left(t\right)=A_{r}\sin\left(2\pi f_{r}t\right)+A_{h}\sin\left(2\pi f_{h}t\right)$.

The above analysis indicates that for a moving radar system, the phase echo of the human is composed of the radar platform phase shift and the displacement of physiological activity; the phase echo of the stationary target only includes the radar platform phase shift. The aim of the proposed method is to recover the vital signals from the mixed observed signals.

### 3.2. Background Clutter

In the airborne radar detection problem, the interference mainly comes from two aspects: (1) The motion of the UAV platform; (2) Echoes from the background environment. When the environment of the human target is the exposed ground, the background clutter is mainly from other detected objects (stationary objects and ground). In this study, we summarize it as the static background environment. When the environment of the human target is the vegetation covered ground, the propeller rotation and wind will cause the movement of the plant blades. In this paper, we summarize it as the dynamic background environment.

#### 3.2.1. Static Background Environment

**Figure 5:**
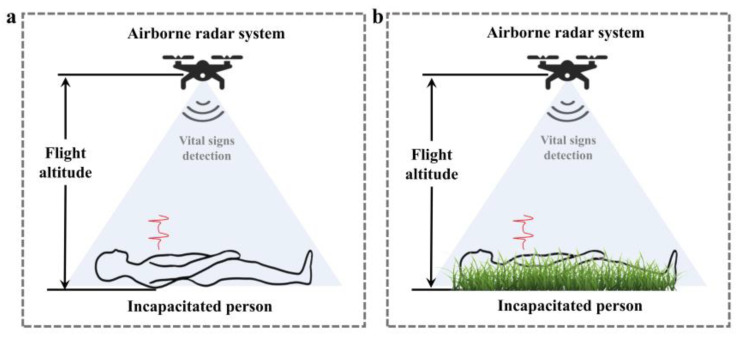
Background environments of the human target: (**a**) Smooth ground. (**b**) Grassland.

Figure 5a is the sketch of the static background environment scenario. Previous studies have observed that echo signals from these static objects are only modulated by the platform motion. It means that the separate measurement of radar platform motion can be inferred from the static clutters in the range profile [20].

Same as Formula (8), the phase signal of a stationary object can be expressed as:

(9) $$\theta_{1}\left(t\right)=\frac{4\pi }{\lambda }d_{UAV}\left(t\right).$$

Assuming that there are several stationary objects in the environment, and the raw echo data can be represented as:

(10) $$R\left(t,\tau \right)=a_{v}s\left(\tau -\tau_{v}\left(t\right)\right)+\sum_{i}a_{si}s\left(\tau -\tau_{si}\left(t\right)\right)+n(t,\tau )$$

where n(t,τ) is noise signal. Representing the above equation in the following matrix forms:

(11) $$R=AS\;R=\left[\begin{array}{c} r_{1}\left(t,\tau \right)\\ r_{2}\left(t,\tau \right)\\ \cdots \\ r_{a}\left(t,\tau \right) \end{array}\right]A=\left[\begin{array}{c} a_{11}\;a_{12}\\ {\;a}_{21}\;a_{22}\;\\ \cdots \\ a_{a1}\;a_{a2} \end{array}\right]S=\left[\begin{array}{c} v\left(t,\tau \right)\\ d_{UAV}\left(t,\tau \right) \end{array}\right]$$

where $R\in R^{a\times n}$, is the multi-range channel echo signal matrix, $A\in R^{a\times 2}$ is the weight matrix, $S\in R^{2\times n}$ is the source signal matrix, and $v\left(t,\tau \right)$ is a vital signal.

Based on the above echo model, the problem of detecting human vital signals with the airborne radar system can be described as a source signals estimation problem, which estimates unknown source signals (vital signals and platform motion signals) from the mixed signals.

#### 3.2.2. Dynamic Background Environment

In this study, we take grassland as a typical dynamic background scenario, as shown in Figure 5b. The grassland scenario has a layered structure. When electromagnetic waves irradiate this rough surface, its scattered waves are no longer plane waves [26]. Assuming that the scattered echo of a target point in the grass is e, the scattered echo of a range bin can be expressed as:

(12) $$E_{i}(t,\tau )=\sum_{j=1}^{n_{g}}e_{j}(t,\tau )$$

where $n_{g}$ is the number of scatter points.

In addition, the propeller rotation will cause the movement of the grass blades, then the grass movement signal of i range bin is given as:

(13) $$N_{i}\left(t,\tau \right)=\sum_{j=1}^{n_{gm}}n_{j}(t,\tau )$$

where $n_{j}(t,\tau )$ is the motion signal of a blade, $n_{gm}$ is the number of the blades.

When the human target is also in this range bin, the radar echo signal phase shift of this range bin can be represented as:

(14) $$\theta \left(t,\tau \right)=\frac{4\pi }{\lambda }\left(r\left(t,\tau \right)+d_{UAV}\left(t,\tau \right)+E_{i}\left(t,\tau \right)+N_{i}\left(t,\tau \right)\right)$$

In this scenario, to accurately extract target information, it is necessary to address two issues: platform motion interference and background clutter interference.

#### 3.2.3. Statistical Characteristic Analysis of Measured Grass-Surface Clutter

It has been observed that the influence of surface conditions in the form of vegetation and roughness will significantly degrade the radar detection performance. Hence, the comprehensive understanding of the clutter statistical characterization is essential for the design of signal processing techniques.

**Figure 6:**
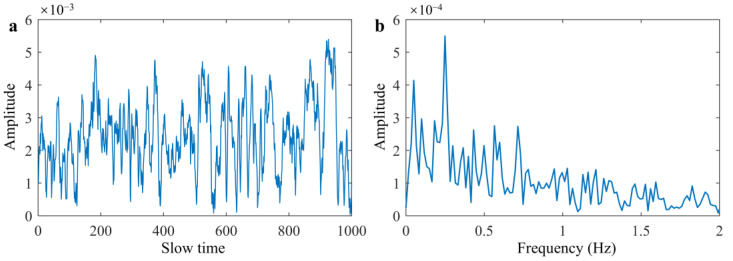
Grass–surface clutter signal. (**a**) Time domain waveform. (**b**) Spectrum.

**Figure 7:**
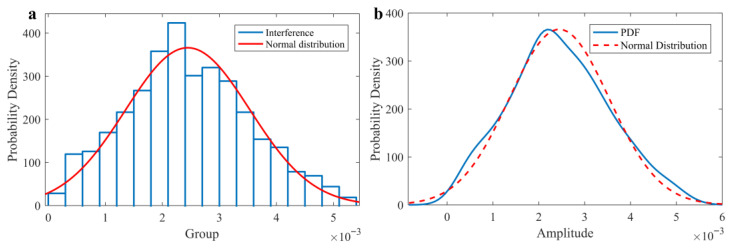
Statistical characteristics of clutter. (**a**) Frequency distribution histogram. (**b**) Fit curve, the probability density function is blue, and the normal distribution fitting curve is red.

In this section, we have taken observations and analysis of grass clutter by the experimental data processing method, which has been the main way to analyze the statistical characteristics of surface clutter [27]. The time domain waveform and spectrum of grassland clutter signal measured by airborne radar are shown in Figure 6, which has the same frequency range as respiration and heartbeat. The frequency distribution histogram of clutter is shown in Figure 7a. It can be observed that its distribution exhibits the unimodal characteristic, which can be attempted to fit a normal distribution curve.

First, the Anderson-Darling test is used to test the normality of the overall distribution of clutter data, and the obtained *p*-value is equal to 0.1655. It indicates that there is no significant difference between the distribution of clutter data and the normal distribution. Then, the analysis looking for the best fit of the clutter signal amplitude probability density function (PDF) is performed, and the regression equation is calculated according to the nonlinear least squares method. The determination coefficient of the regression model is equal to 0.9873, ensuring the effect and accuracy of the fitting result.

The results indicate that the clutter signal may obey the Gaussian distribution or the Gaussian mixture distribution, which could not be filtered by preprocessing. According to the result, the use of high-order cumulants as analytical tools theoretically can completely suppress the influence of Gaussian noise because the higher-order cumulants of Gaussian processes are always equal to zero.

## 4. Proposed Vital Signals Extraction Method

**Figure 8:**
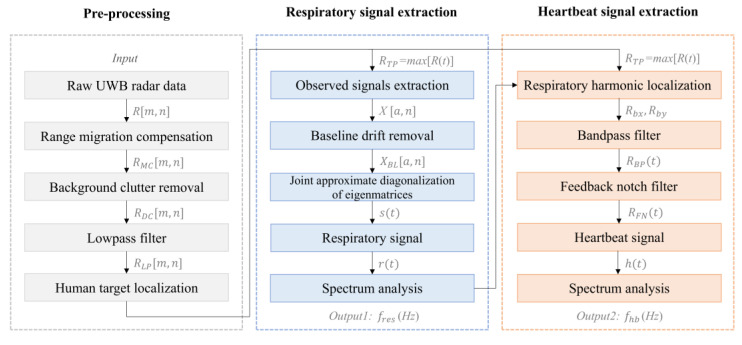
The block diagram of the vital signal extraction method.

Human vital signals include respiration and heartbeat, which implicate the life state of the human body. The overall process of the proposed vital signals extraction method is shown in Figure 8, which mainly includes pre-processing, respiratory signal extraction, and heartbeat signal extraction.

### 4.1. Pre-Processing

#### 4.1.1. Range Migration Compensation

**Figure 9:**
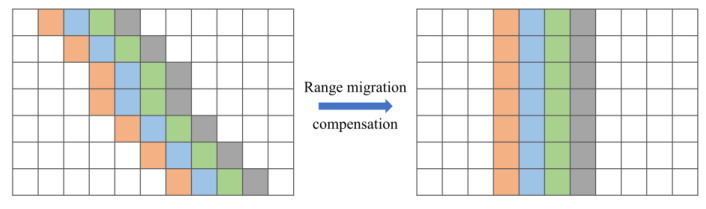
Schematic diagram of range migration compensation.

The drone hovers above the human target to be detected, and the radar continuously transmits electromagnetic waves to extract vital signs from the radar echo sequences. During this process, the drone will generate relative movements, with distance variation between the array antenna and the target. The position of the target point in the echo will be changed, resulting in range migration between radar echo sequences. The aim of range migration compensation is to correct this offset, which includes envelope alignment and phase compensation. Figure 9 shows the schematic diagram of range migration compensation.

Envelope alignment is the process of translating the echo data to eliminate distance misalignment, also known as coarse compensation. For two adjacent echo sequences, the distance between the target and the radar changes very little, and their envelopes have a strong correlation. Based on this, the adjacent envelope correlation method calculates the corresponding delay through peak values of their cross-correlation function, which can realize alignment on range bins [28]. Assuming that $r_{1}(t)$ and $r_{2}(t)$ are signal sequences of adjacent range with strong correlation, their cross-correlation function is:

(15) $$R_{12}(∆t)=\int r_{1}(t)r_{2}(t-∆t)d∆t$$

The displacement amount can be estimated from the maximum position of the cross-correlation function:

(16) $$\tau_{12}=argmax(R_{12}\left(∆t\right))$$

Then, shifting the corresponding signal according to the value, the range alignment of these two signals can be completed. The above operation should then be repeated until all echo signals are aligned in range.

**Figure 10:**
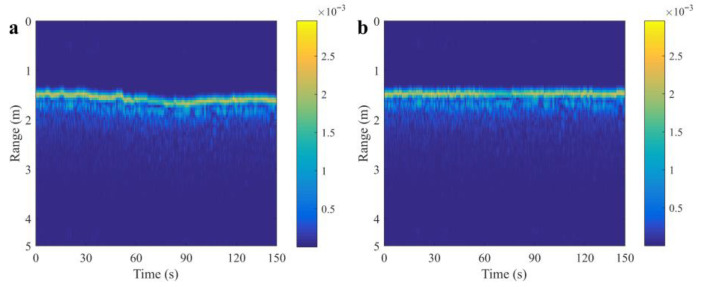
Field test result. (**a**) Raw data. (**b**) After compensation.

After completing envelope alignment, the next step is the phase compensation, also known as fine compensation. Taking the weighted average of the first $N_{a}$ envelope-aligned echoes as the benchmark, the average value of the difference between the phase of the echo signal and the benchmark is the phase compensation value of this echo signal. Figure 10 illustrates the example after range migration compensation processing.

#### 4.1.2. Background Clutter Removal

**Figure 11:**
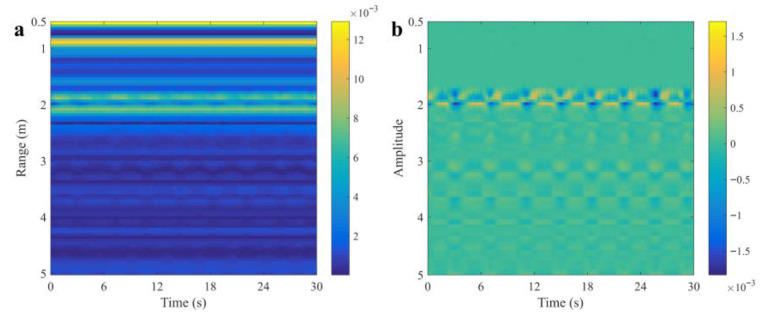
Two-dimensional pseudo-color image of UWB radar. (**a**) Raw radar data. (**b**) Data after preprocessing.

**Figure 12:**
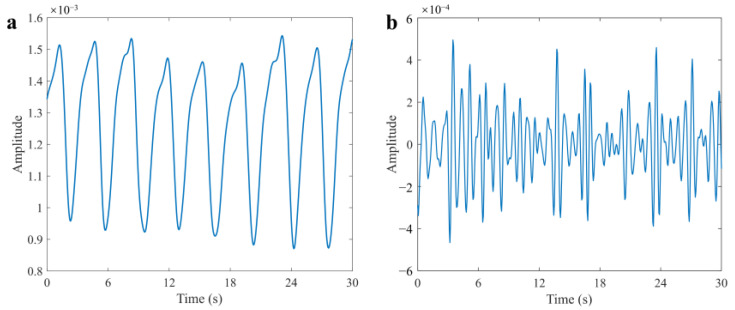
Extracted vital signals at the target position. (**a**) Respiratory signal. (**b**) Heartbeat signal.

The raw radar data contains direct-current (DC) components caused by static objects and baseline drift caused by environmental factors. These two types of noise are known as background clutter, which will cause strong interference to the wanted signal. The adopted 100 order DC drift removal method is represented by the following equation:

(17) $$R_{DC}\left(m,\;n\right)=R_{MC}\left(m,n\right)-\frac{1}{100}R_{MC}\left(m,n\right)$$

where $R_{DC}\left(m,\;n\right)$ is the radar data after processing and $R_{MC}\left(m,n\right)$ is the data after range migration compensation. The 2D pseudo-color image of the raw UWB radar data and the data after preprocessing are displayed in Figure 11. The measurement was conducted indoors with the radar fixed at 2 m above a human target lying on the ground. The respiratory and heartbeat signals can be obtained by extracting the echo signals of the corresponding range bin and performing band-pass filtering, and the waveforms are displayed in Figure 12.

#### 4.1.3. Human Target Localization

After the above processing, background interference has been removed, and it is necessary to locate and select the optimal range unit, which is the location of the human target. Computing the square sum of the slow time, the range unit with the maximum sum is the position of the target.

(18) $$S\left(j\right)=\sum_{i=1}^{n}{R^{2}}_{DC}(j,i)\;R_{TP}=max{\left[S\left(j\right)\right]}_{j=1}^{j=m}$$

where $R_{DC}(j,i)$ is the slow time signal of one range, $S\left(j\right)$ is the accumulation of $R_{mc}(j,i)$, and $R_{TP}$ is the range bin with the biggest accumulation.

### 4.2. Respiratory Signal Extraction

#### 4.2.1. Observed Signals Extraction

Pulse radar divides the entire detection range into equidistant bins, each of which is also known as a range gate. The distributed scatter points on the thorax of a human target can extend over several centimeters of range and appear on the multiple range gates around the range gate with the biggest accumulation [24]. The probable solution of this problem is to extract the upper and lower two range gates centered on the energy maximum range gate, and five channel signals are extracted in total. Then, the BEADS algorithm is adopted to remove baseline drift [29].

#### 4.2.2. Joint Approximate Diagonalization of Eigenmatrices Algorithm

According to Section 3, the airborne radar vital signals detection problem can be described as a source signal separation problem. The source signals are respiratory signal, heartbeat signal, platform motion, and interference from the environment, and the observed signals are instantaneous linear mixture of the source signals. From Equation (11), it can be concluded that the observed signals can be written as:

(19) $$r_{1}\left(t\right)=a_{11}s_{1}\left(t\right)+a_{12}s_{2}\left(t\right)+\cdots +a_{1t}s_{t}\left(t\right)\;r_{2}\left(t\right)=a_{21}s_{1}\left(t\right)+a_{22}s_{2}\left(t\right)+\cdots +a_{2t}s_{t}\left(t\right)\;\vdots \;r_{a}\left(t\right)=a_{a1}s_{1}\left(t\right)+a_{a2}s_{2}\left(t\right)+\cdots +a_{at}s_{t}\left(t\right)$$

The observed signals can be modeled in the following way [30]:

(20) R=AS

where R is the data matrix, A is the mixing matrix, and S is the source component matrix.

Blind source separation JADE algorithm first whitens the observed signals, the whitening matrix V satisfies [31]:

(21) VA=U

where U is the unitary matrix to be calculated. The covariance matrix of the observed data is:

(22) $$R_{rr}=E[R(k){R(k)}^{H}]$$

Assuming there is no noise, then $R_{rr}$ is not full rank, and it has M non-zero eigenvalues $\mu_{1},\mu_{2},\;{\cdots ,\;\mu }_{m}$, $g_{1},\;g_{2},\;{\cdots ,g}_{m}$ are corresponding feature vectors, the whitening matrix can be expressed as:

(23) $$V={\left({\mu_{1}}^{-\frac{1}{2}}g_{1},{\mu_{2}}^{-\frac{1}{2}}g_{2},\cdots ,{\mu_{m}}^{-\frac{1}{2}}g_{m}\right)}^{T}$$

When there is additive Gaussian white noise, $R_{rr}$ is generally full rank, arranging M feature vectors in descending order: $g_{1}\geq g_{2}\geq \cdots \geq g_{m}\geq g_{m+1}\geq \cdots \geq g_{n}$, the variance of noise can be written as:

(24) $$\sigma^{2}=\frac{1}{n-m}\sum_{i=1}^{n-m}g_{m+i}$$

(25) $$V={\left[({\mu_{1}-\sigma^{2})}^{-\frac{1}{2}}g_{1},{{(\mu }_{2}-\sigma^{2})}^{-\frac{1}{2}}g_{2},\cdots ,{\left(\mu_{m}-\sigma^{2}\right)}^{-\frac{1}{2}}g_{m}\right]}^{T}$$

The whited signal can be represented as:

(26) $$\tilde{r}\left(k\right)=VR\left(k\right)=V\left[AS\left(k\right)+N\left(k\right)\right]=US\left(k\right)+VN(k)$$

The fact that the higher-order cumulants of any Gaussian process are equal to 0 makes it theoretically possible to completely suppress the impact of Gaussian noise. To separate the wanted signal through the subsequent blind source separation algorithm, it is necessary to construct a fourth-order cumulant matrix of the whitened signal and perform eigenvalue decomposition to obtain the unitary matrix.

Denoting the i column of the unitary matrix is $u_{i}$, $U=u_{1},u_{2},\cdots ,u_{i},\cdots ,u_{m}$ and $u_{i}={\left(u_{i1},u_{i2},{\cdots ,u}_{im}\right)}^{T}$. Define $M_{i}$ as follows:

(27) $$M_{i}=u_{i}{u_{i}}^{T},i=1,2,\cdots ,m$$

where the kl-th element of $M_{i}$ is $m_{i,kl}=u_{ik}u_{il}$.

The fourth-order cumulant matrix is defined as [32]:

(28) $$Q_{\tilde{r}}\left(M_{i}\right)=\sum_{k,l=1,n}cum({\tilde{r}}_{p},{\tilde{r}}_{q},{\tilde{r}}_{k},r_{l})m_{i,lk},1\leq p,q\leq n$$

Performing eigenvalue decomposition on the fourth-order cumulant matrix $Q_{\tilde{r}}\left(M\right)$, the estimated matrix $\tilde{U}$ of the unitary matrix U can be obtained:

(29) $$Q_{\tilde{r}}\left(M\right)=\tilde{U}\Lambda {\tilde{U}}^{T}$$

(30) $$\Lambda =Diag[\mu_{1}u_{1}M{u_{1}}^{T},\mu_{2}u_{2}M{u_{2}}^{T},\cdots ,\mu_{m}u_{m}M{u_{m}}^{T}]$$

Finally, the estimated signals can be obtained by the estimated matrix:

(31) $$Y\left(k\right)={\tilde{U}}^{-1}\tilde{r}={\tilde{U}}^{-1}VR$$

The specific process of JADE algorithm is as follows:

(1) Decentralizing and whitening the observed signals matrix;
(2) Constructing the high-order cumulant matrix $Q_{\tilde{r}}$ of the whited matrix;
(3) Performing joint approximate diagonalization on the matrix $Q_{\tilde{r}}$ to obtain the estimated matrix $\tilde{U}$ of the unitary matrix U;
(4) Estimating the source signal according to Equation (31).

### 4.3. Heartbeat Signal Extraction

#### 4.3.1. Bandpass Filter

After the above analysis, interference is mainly concentrated in low frequency range, and the heart rate range of a normal person is 0.85–3.3 Hz. Hence, the lower cutoff frequency of the bandpass filter is 0.85 Hz, and the upper cutoff frequency of the bandpass filter is 3.3 Hz. The processed signal $R_{BP}\left(t\right)$ can be represented as:

(32) $$R_{BP}\left(t\right)=R_{TP}(t)*H_{BP}(t)$$

where $H_{BP}(t)$ is the impulse response function of the bandpass filter.

#### 4.3.2. Respiratory Harmonic Localization

After obtaining the fundamental frequency of the respiratory signal, locate its third, fourth, and fifth harmonics:

(33) $$R_{FLP}=\int_{-\infty }^{+\infty }r(t)e^{-j\omega t}dt\;R_{bx},R_{by}=\max\;value(R_{FLP})\;R_{h}=kR_{bx}$$

where r(t) is the extracted respiratory signal in Section 4.2.2, $R_{bx}$ is the respiratory fundamental frequency, and $R_{by}$ is amplitude of $R_{bx}$, k=3, 4, 5.

#### 4.3.3. Feedback Notch Filter

The notch filter is given by the following equation:

(34) $$G\left(z\right)=\frac{1-2\cos\left(\omega^{*}\right)z^{-1}+z^{-2}}{1-2\rho \cos\left(\omega^{*}\right)z^{-1}+{\rho^{2}z}^{-2}}$$

where $\omega^{*}$ is the notch frequency, ρ is the radius of the poles of $G\left(z\right)$.

**Figure 13:**
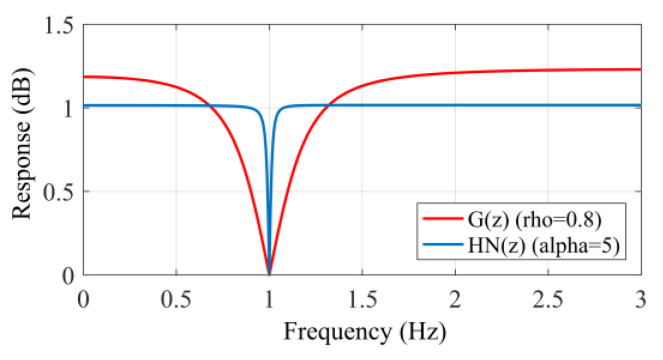
Amplitude–frequency curve of the feedback notch filter.

Introducing the feedback structure on traditional notch filter [33], and the transfer function can be expressed as:

(35) $$H_{N}\left(z\right)=\frac{(1+\alpha )G\left(z\right)}{1+\alpha G\left(z\right)}$$

where α is the feedback coefficient. As shown in Figure 13, the addition of the feedback structure makes the overshoot and bandwidth of the notch filter achieve the ideal degree, improving the performance of the notch filter. The feedback notch filter is utilized to remove the third, fourth, and fifth respiratory harmonics.

## 5. Experiment and Results

### 5.1. Experimental Setup

**Figure 14:**
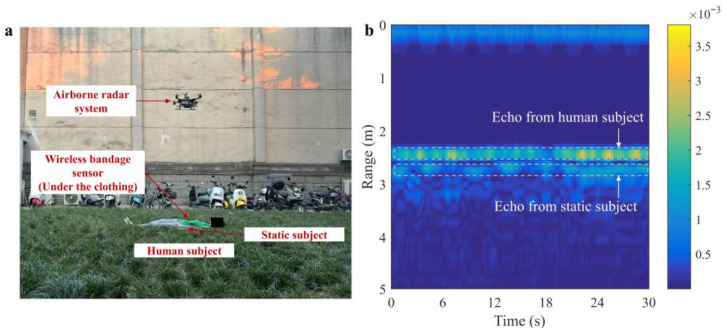
Experiment in scenario 1. (**a**) Scenario setup. (**b**) Raw radar data.

Two field experiments in different scenarios are carried out to test and verify the proposed method, and the experimental setups are shown in Figure 14a. Scenario 1 is grassland, and scenario 2 is wall penetration. The human subject lies in the detection area with normal breathing as the test target, facing the radar transceiver antenna. The measurement time is 30 s.

The parameter setup of X4M200 UWB radar is listed in Table 1. To evaluate experimental result, the subject also wears ErgoLab contact bandage sensor, which could wirelessly collect respiratory and electrocardiogram (ECG) signals. The physiological signals collected by the contact sensor are regarded as the ground truth. Finally, we adopt the existing radar self-motion cancellation method in [19] for comparison. The reference method compensates for the platform motion by calculating residual phase between the human subject and the static clutter, and then it decomposes the residual for vital signals extraction. In our experiments, we place a metal reflector plate in the environment as the stationary object.

In addition, the signal-to-noise ratio (SNR) is used to evaluate whether the proposed method can accurately estimate the respiratory rate (RR) and the heartbeat rate (HR). The definition of SNR is:

(36) $$SNR=10{log}_{10}\frac{{\left|S(k_{max})\right|}^{2}}{\frac{1}{N-1}(\sum_{k=0}^{k_{max}-1}{\left|S(k)\right|}^{2}+\sum_{k=k_{max}+1}^{N}{\left|S(k)\right|}^{2})}$$

where S(k) is the signal spectrum, N is the number of sampling points of the spectrum, and $k_{max}$ is the peak coordinate of the spectrum. Considering that the respiratory and heartbeat spectrum are generally between 0 Hz and 2 Hz, we only examine the frequency information within this frequency band.

### 5.2. Performance in Realistic Grassland Scenario

**Figure 15:**
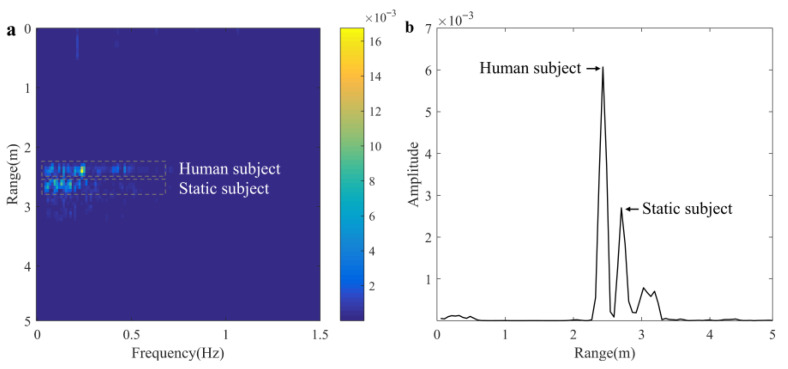
Target location. (**a**) Range FFT of raw data. (**b**) Cross-section view.

**Figure 16:**
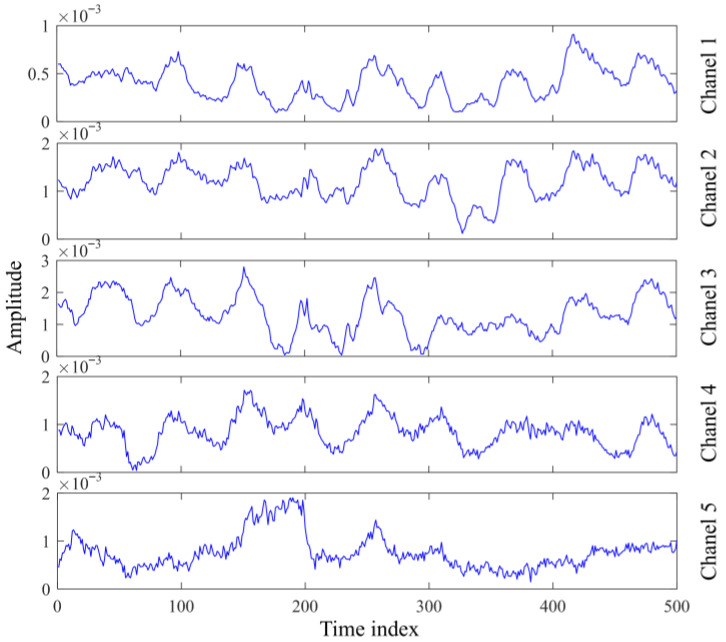
Observed signals.

The experimental scenario setup is shown in Figure 14a, the unprocessed radar echo data in this scenario are shown in Figure 14b, and the target localization result is shown in Figure 15. The echo of the human target is clear and visible, while the echo of the reflector is fuzzy, which is due to range ambiguities. The observed signals of extracted five range gates are shown in Figure 16, which are similar in waveform but differ in amplitude, and the signal of the middle range unit has the largest energy. The observed signals for each range bin can be considered as the linear combination of cardiac signal, chest motion, platform motion, and noise. In this experiment, the 46^th^-to-50^th^-range bins are identified and selected for extracting the vital signals.

**Figure 17:**
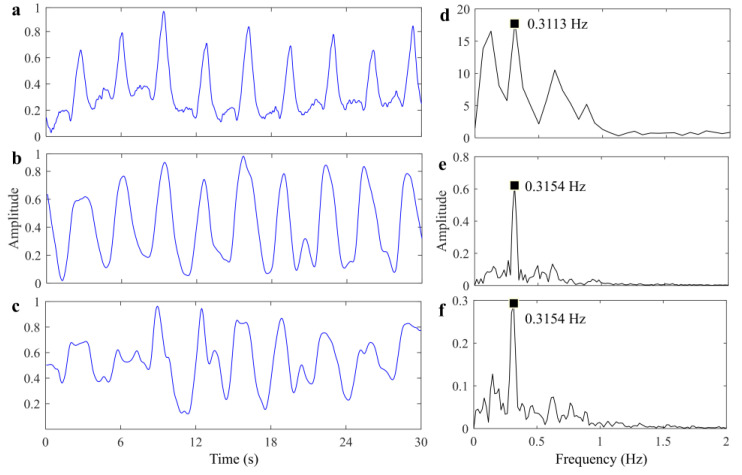
Results of scenario 1. (**a**) Reference respiratory signal. (**b**) Recovered respiratory signal by proposed method. (**c**) Recovered respiratory signal by reference method. (**d**) Spectrum of signal (**a**). (**e**) Spectrum of signal (**b**). (**f**) Spectrum of signal (**c**).

After acquisition of raw radar data, signal processing method mentioned in Section 4 is implemented. The respiratory signal extraction results are expressed in Figure 17. According to the corresponding spectrum, it can be inferred that the RR of the bandage sensor is 0.3113 Hz, the RR of the proposed method is 0.3154 Hz, and the RR of the reference method is also 0.3154 Hz. The accuracy of UWB radar is 98.68%, and the difference is caused by time interval error and systematic error, which can be ignored generally. Figure 17b,c clearly shows that the respiratory signal extracted by the proposed method is closer to the reference waveform and has higher SNR compared with the signal extracted by the reference method. The effectiveness of the reference method depends on the quality of the echo signal of the stationary object. In addition, this method requires one or more stationary objects as the reference signal to recover the vital signals, while such restrictive conditions may result in performance degradation in a more cluttered environment.

**Figure 18:**
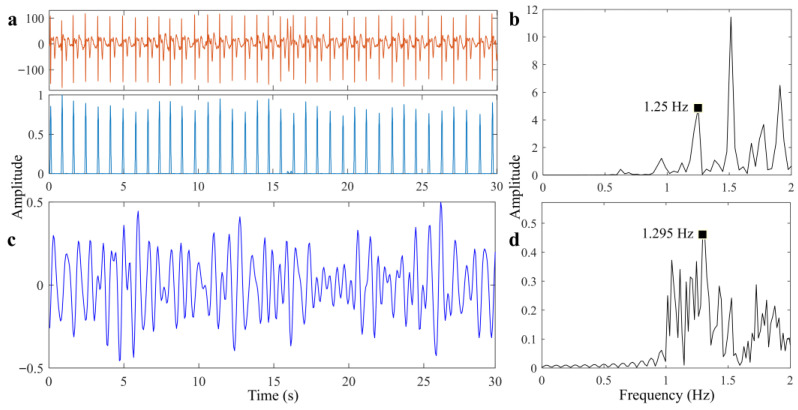
Results of scenario 1. (**a**) Reference heartbeat signal. (**b**) Spectrum of signal (**a**). (**c**) Recovered heartbeat signal. (**d**) Spectrum of signal (**c**).

The results of heartbeat signal extraction are shown in Figure 18. Figure 18a is the reference ECG data, and Figure 18c is the extracted heartbeat signal after removing the fifth-order respiratory harmonic. The acquired reference HR according to the peak detection method is 1.25 Hz, the estimated HR of Radar is 1.295 Hz, and the accuracy is 96.4%. Comparison of detection results indicates that the proposed method is able to detect the overall movement information of the heart, while the attenuation of morphological amplitude is significant, and this is in line with our expected estimation.

The results in the first scenario suggest that the proposed method can effectively suppress background clutter and noise interference from the environment, recover the respiratory and heartbeat signals, and improve the SNR.

### 5.3. Performance in Through-the-Wall Scenario

**Figure 19:**
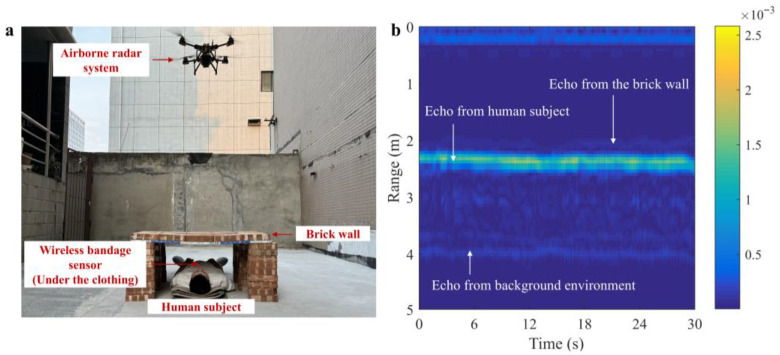
Experiment in scenario 2. (**a**) Scenario setup. (**b**) Raw radar data.

**Figure 20:**
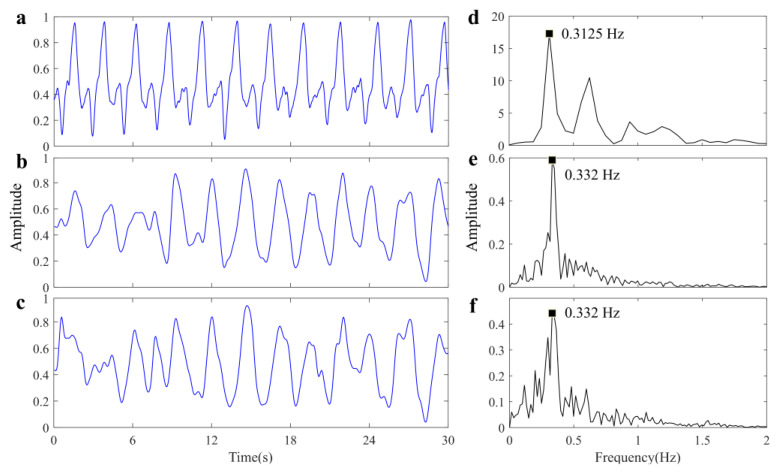
Results of scenario 2. (**a**) Reference respiratory signal. (**b**) Recovered respiratory signal by proposed method. (**c**) Recovered respiratory signal by reference method. (**d**) Spectrum of signal (**a**). (**e**) Spectrum of signal (**b**). (**f**) Spectrum of signal (**c**).

For comprehensive verification of the proposed method, we also explore the feasibility of utilizing the airborne radar system for monitoring the vital signals of a single subject through a brick wall. Figure 19 illustrates the experimental scenario description and the collected radar data. The extracted respiratory signal is shown in Figure 20. It has been observed that the captured RR of the bandage sensor is 0.3125 Hz, the RR of the proposed method is 0.332 Hz, and the RR of the reference method is also 0.332 Hz. The accuracy of UWB radar is 93.76%, and the system’s performance experiences slight degradation due to the inevitable attenuation effect of obstacles on electromagnetic waves. Table 2 presents a quantitative comparison between the proposed method and the reference method in two scenarios, and the results demonstrate the superior performance and robustness of our proposed method in respiratory waveform recovery.

Under wall-penetration detection conditions, the bio-radar echo of human physiological motion is weak, and attenuation will reduce the power of the echo, causing distortion of the echo. In addition, multipath effects will seriously affect effective micro-Doppler feature detection and separation. Reducing the center frequency of the radar can enhance its penetration capability, but the sensitivity of the radar will also decrease accordingly. Hence, through-the-wall independent heartbeat signal isolation from the mixture is also challenging for ideal environments [34,35].

### 5.4. Impact of Distance Between the System and the Victim

Considering that the distance between the victim and the airborne radar system is not fixed in practical applications, we also investigate whether the distance would impact the performance of the system in scenario 1. The detection distance ranges from 2 to 5 m. Table 3 shows the results of the estimation performance of RR and HR for different distances. It illustrates that the estimation performance degrades as the detection distance increases due to the energy attenuation of radar echo signal, which can be addressed by increasing transmission power.

## 6. Conclusions

In this paper, an airborne IR-UWB radar system for vital signs detection of the unconscious victim has been developed. The communication distance of the developed system is up to 10 km with low time delay and low packet loss rate, which satisfies the real-time requirement of remote vital signs monitor. In addition, a novel framework based on blind source separation for precise respiration and heartbeat extraction has been proposed. To resolve the signal distortion raised by the background environments, we analyze the statistical characteristics of measured grass clutter and note that the radar echo of clutter obeys the Gaussian distribution. Then, a signal processing framework based on the JADE algorithm is proposed to estimate the respiratory and heartbeat signals from the mixed signals. Extensive results in field trials demonstrate the effectiveness and accuracy of the proposed method. This work may provide a new solution for intelligent SAR in harsh environments.

## Figures and Tables

**Table 1:** Radar experimental parameters.

Center Frequency	Bandwidth	Detection Zone	Range Resolution	Frame Rate
7.29 GHz	1.4 GHz	0.4~5 m	0.0514 m	17 Hz

**Table 2:** Results of respiratory rate estimation.

Parameter	RR (Hz)			Accuracy (%)		SNR (dB)	
	Reference	Proposed Method	Reference Method	Proposed Method	Reference Method	Proposed Method	Reference Method
Scenario 1	0.3113	0.3154	0.3154	98.68	98.68	12.457	11.035
Scenario 2	0.3125	0.332	0.332	93.76	93.76	9.002	7.983

**Table 3:** Results of different distances.

Parameter	RR (Hz)	Accuracy (%)	HR (Hz)	Accuracy (%)
2 m	0.2366	98.50	1.017	98.44
3 m	0.3113	96.48	1.166	96.25
4 m	0.2449	95.74	1.137	95.99
5 m	0.2813	93.15	1.148	90.22

## Data Availability

The original contributions presented in this study are included in the article. Further inquiries can be directed to the corresponding authors.
